# Die Grundsicherung für Arbeitsuchende im europäischen Kontext

**DOI:** 10.1007/s10273-021-3006-2

**Published:** 2021-09-21

**Authors:** Regina Konle-Seidl

**Affiliations:** Migration, Integration und internationale Arbeitsmarktforschung, Institut für Arbeitsmarkt-und Berufsforschung (IAB), Regensburger Str. 104, 90478 Nürnberg, Deutschland

**Keywords:** I32, I,38, I 39, J08

## Abstract

Während auf europäischer Ebene eine Stärkung von Mindestsicherungssystemen im Rahmen der Umsetzung der Europäischen Säule sozialer Rechte diskutiert wird, ist die deutsche Diskussion im Vorfeld der Bundestagswahl 2021 durch unterschiedlich weitreichende Vorschläge zur Reform des Grundsicherungssystems (Überwindung von Hartz IV) geprägt. Die auf EU-Ebene verwendeten Kriterien zur Beurteilung von Mindestsicherungssystemen werden beschrieben, und die deutsche Grundsicherung wird in den europäischen Benchmarking-Rahmen eingeordnet. Vor diesem Hintergrund werden Vorschläge zur Reform des Harz-IV-Systems aufgezeigt.

## Abstract (en)

While the European Commission envisages a strengthening of minimum income schemes as part of the implementation of the European Pillar of Social Rights, the political debate in Germany is characterised by proposals of varying scope for reforming the basic income system for jobseekers (“Hartz IV”). This article places the German basic income system in the European context and discusses its role and performance from a European perspective. Against this background, the article also discusses proposals for reforming the “Harz IV system”.

Nach dem Subsidiaritätsprinzip liegt die Verantwortung für die soziale Mindestsicherung in der Europäischen Union grundsätzlich bei den Mitgliedstaaten. Strategien zur Bekämpfung von Armut und sozialer Ausgrenzung sind aber integraler Bestandteil der europäischen Beschäftigungs- und Sozialpolitik. Unter der deutschen EU-Ratspräsidentschaft verabschiedete der Europäische Rat im Oktober 2020 Schlussfolgerungen zur „Stärkung der Mindesteinkommenssicherung zur Bekämpfung von Armut und sozialer Ausgrenzung in der COVID-19-Pandemie und darüber hinaus". Der Rat lädt die EU-Kommission ein, Vorschläge für einen neuen Unionsrahmen zur effektiven Unterstützung nationaler Mindestsicherungssysteme bis Ende 2022 auszuarbeiten.

## Kennzeichen von Mindestsicherungssystemen

Das erklärte Ziel von Mindestsicherungssystemen ist die Sicherung des Lebensunterhalts, also der Schutz vor Armut. Es gibt jedoch keine allgemeingültige Definition von Mindestsicherungssystemen, und die Vielfalt von bedürftigkeitsgeprüften Last-Resort-Sicherungssystemen in Europa ist groß. Ganz allgemein bezeichnet die soziale Mindestsicherung den Teil des Sozialsystems, der mit der Bekämpfung von Armut und sozialer Ausgrenzung betraut ist und grundsätzlich einer mehr oder weniger breit definierten hilfsbedürftigen Bevölkerung einen Mindesteinkommensstandard zusichert. Ein gemeinsames Kennzeichen von Mindest- und Grundsicherungssystemen ist, dass sie Leistungen gewähren, die nicht an eine frühere Beschäftigung oder eine Beitragshistorie gebunden sind und eine Bedürftigkeitsprüfung als zentrales Anspruchskriterium verwenden. Sie stellen einen steuerfinanzierten Einkommensersatz für Erwerbsfähige dar und stocken in der Regel (unzureichende) Leistungen aus der Arbeitslosenversicherung auf. Hierzulande gewährt die SGB-II-Grundsicherung auch Beschäftigten aufstockende Leistungen in Form eines In-Work Benefit, wenn das Einkommen aus Erwerbsarbeit das „soziokulturelle" Existenzminimum im Haushaltskontext unterschreitet. Neben der SGB-II-Grundsicherung für Arbeitsuchende gibt es zwei weitere bedürftigkeitsgeprüfte Mindestsicherungsleistungen: Die „Grundsicherung für Erwerbsunfähige und im Alter" für erwerbsunfähige Personen und für Rentner:innen mit einer Rente unterhalb des Existenzminimums sowie „Hilfe zum Lebensunterhalt" (Sozialhilfe) für Menschen in stationären Einrichtungen.

Sowohl bei der Definition der Armutsschwelle, den Zugangsregeln zu Mindestsicherungsleistungen (Bedarfsprüfung), der Berechnung der Regelsätze als auch dem Angebot an aktiven Eingliederungsleistungen und den mit dem Leistungsbezug verbundenen Pflichten (Fördern und Fordern) unterscheiden sich die europäischen Wohlfahrtsstaaten aber erheblich voneinander.

## Besondere Stellung der deutschen Grundsicherung

Als letzte Instanz im Gesamtsystem der sozialen Sicherung eines Landes nehmen die meisten Mindestsicherungssysteme in Europa eine residuale Rolle ein und dienen in

**Regina Konle-Seidl** ist Senior Researcher am Institut für Arbeitsmarkt- und Berufsforschung (IAB) und derzeit an das Europäische Parlament abgeordnet.

erster Linie der Armutsbekämpfung. Daten zur Inzidenz, d.h. dem Anteil aller Hilfeempfangenden an der Bevölkerung im Alter von 18 bis 64 Jahren zeigen, dass in Ländern wie Dänemark, Schweden, Niederlande oder Belgien zwischen 3 % und 4 % der Bevölkerung im erwerbsfähigen Alter auf Mindestsicherungsleistungen angewiesen ist. In Deutschland waren es 2020 7,4 %. Seit der Zusammenlegung der Arbeitslosen- und Sozialhilfe 2005 ist die SGB-II-Grundsicherung nicht nur das letzte soziale Auffangnetz in Deutschland, sondern auch die dominante Sozialleistung bei Bedürftigkeit und Arbeitslosigkeit. Im Jahr 2020 erhielten knapp 60 % aller Arbeitslosen ALG-II-Leistungen. Trotz der (irreführenden) Bezeichnung „Grundsicherung für Arbeitsuchende" werden Leistungen unabhängig vom Arbeitslosenstatus all denjenigen gewährt, die eine bestimmte Einkommensgrenze (soziokulturelles Existenzminimum) bezogen auf alle Haushaltsmitglieder unterschreiten. Die SGB-Grundsicherung ist damit umfassender als die meisten anderen Mindestsicherungssysteme in Europa und nur noch vergleichbar mit dem britischen Universal Credit, der rund 9 % der Wohnbevölkerung sozial absichert.

Zuvorderst dient die Grundsicherung für Arbeitsuchende der Existenzsicherung erwerbsfähiger Leistungsberechtigter und weiterer Personen in deren Bedarfsgemeinschaft. Um ein Leben zu ermöglichen, das der Würde des Menschen entspricht, erhebt sie aber neben der materiellen Versorgung breiter Schichten der Bevölkerung mehr als die meisten Mindestsicherungssysteme in Europa den Anspruch, soziale und kulturelle Teilhabe zu gewährleisten. Gleichzeitig hat sie zum Ziel, die Menschen zu befähigen, wieder selbst für sich zu sorgen, bestenfalls über die Aufnahme einer bedarfsdeckenden Beschäftigung. Hierzu wird z.B. in die Erhöhung der Beschäftigungsfähigkeit investiert oder durch arbeitsmarktpolitische Maßnahmen bei der Aufnahme einer Erwerbstätigkeit unterstützt.

Die große Reichweite der Grundsicherung für Arbeitsuchende (Hartz IV) ist zum einen durch die Abschaffung der lohnbezogenen Arbeitslosenhilfe zu erklären. In anderen Ländern, in denen es keine solche Anschluss- oder Zwischenleistung wie die Notstandshilfe in Österreich oder das Basisarbeitslosengeld in Finnland gibt, sind vorgelagerte Versicherungssysteme im Fall von Arbeitslosigkeit oder Erwerbsunfähigkeit wichtiger. Die Reichweite von Versicherungsleistungen bei Arbeitslosigkeit oder Erwerbsunfähigkeit hängt wiederum von den Anspruchsvoraussetzungen ab. Da hierzulande Rahmenfristen und Anwartschaftszeiten in der Arbeitslosenversicherung im Ländervergleich relativ strikt, die maximale Bezugsdauer relativ kurz und der Zugang zu Erwerbsunfähigkeitsleistungen schwierig ist, sind mehr Menschen in der Grundsicherung als in vergleichbaren Wohlfahrtsstaaten. Dazu kommt, dass „Erwerbsfähigkeit" in Deutschland sehr breit definiert wird und somit viele Menschen mit sozialen und gesundheitlichen Einschränkungen SGB-II-Leistungen beziehen (Konle-Seidl et al., 2014).

## Zwischenstaatlicher Vergleich durch komplexe Regelungen erschwert

Die Leistungsfähigkeit, also die Fähigkeit von Mindestsicherungssystemen vor Armut zu schützen, hängt nicht nur von der Großzügigkeit und der Verfügbarkeit von Mindestsicherungsleistungen im Bedarfsfall ab, sondern auch von national festgelegten Bedürftigkeits- bzw. Armutsgrenzen. Die einzelnen Mitgliedsländer legen diese Schwelle z.B. auf Basis eines Warenkorbs, den Lebenshaltungskosten von Haushalten mit niedrigem Einkommen (Deutschland), als Anteil am Mindestlohn (Niederlande) oder Arbeitslosengeld (Dänemark) fest. Komplexe und/oder restriktive Regeln können hierbei zu Ausschlussfehlern oder Nichtinanspruchnahme von Leistungen (Non-Take-Up[1]) führen und letztlich dazu, dass das System sehr selektiv ist und vulnerable Gruppen wie z.B. Obdachlose oder Migrant:innen erst gar nicht erreicht.

Weiterhin gibt es in einer Reihe von Ländern alters- und aufenthaltsbezogene Einschränkungen im Zugang zu Mindestsicherungsleistungen. In Frankreich sind junge Menschen (ohne Kinder) erst ab 25 Jahren förderfähig, wenn sie zuvor nicht innerhalb der vergangenen drei Jahre mindestens zwei Jahre Vollzeit gearbeitet haben. In den Niederlanden hat für unter 27-Jährige eine Ausbildung immer Vorrang vor Sozialhilfe, und in Dänemark erhalten unter 30-Jährige geringere Leistungen als über 30-Jährige. Zudem gibt es in Dänemark oder Italien Mindestsicherungsleistungen für Ausländer:innen erst nach neun bzw. zehn Jahren Aufenthalt. Weiterhin bekommen in Dänemark Drittstaatsangehörige geringere Leistungen. Eine weitere Besonderheit der Sozialhilfe in Dänemark ist, dass sie als Individualleistung gewährt wird, sodass in Paarhaushalten (ohne Kinder) die Regelleistung für eine Person vollständig entfällt, wenn eine arbeitsfähige Person in den vorangegangenen zwölf Monaten nicht mindestens 225 Stunden gearbeitet hat. In der rechtsbasierten deutschen Grundsicherung ist der Zugang dagegen universeller. Es gibt keine Zugangsbeschränkungen im Hinblick auf Alter. Der Zugang zu SGB-II–Leistungen ist neben Asylbewerber:innen im Anerkennungsverfahren nur für EU-Ausländer:innen eingeschränkt, deren Aufenthaltsrecht sich allein aus dem Zweck der Arbeitssuche ergibt.

1 Das IAB schätzt, dass zwischen 34 % und 43 % aller potenziell Anspruchsberechtigten ihren Grundsicherungsanspruch nicht realisieren. Dies reduziert die Effektivität der Grundsicherung. Noch höhere „non-take-up"-Raten gibt es laut Eurofound (2015) in Österreich (49 % bis 62 %, 2003), Belgien (57 % bis 75 %, 2005), Finnland (rund 50 %, 2010) und Portugal (72 %, 2001).

## Benchmarking auf EU-Ebene

In den integrierten EU-Leitlinien für die Beschäftigungs- und Sozialpolitik sind Maßnahmen zur Bekämpfung von Armut und sozialer Ausgrenzung im Einklang mit dem Ansatz der aktiven Eingliederung festgelegt. Darin sind auch die Elemente der Europäischen Säule sozialer Rechte integriert. Diese Leitlinien bilden die Rechtsgrundlage für den Prozess des Europäischen Semesters, der einen Rahmen für die Überwachung und Koordinierung der Wirtschafts- und Sozialpolitik in der gesamten Europäischen Union bietet. In diesem Rahmen führt die EU-Kommission auch ein Monitoring von EU-Mindestsicherungssystemen durch, unter anderem um den Mitgliedstaaten länderspezifische Empfehlungen an die Hand zu geben. Die Beurteilung der Leistungsfähigkeit von nationalen Mindestsicherungssystemen basiert dabei auf drei zentralen Kriterien: der Angemessenheit von Leistungen, einem universellen Zugang zu Mindestsicherungsleistungen und der Verfügbarkeit von arbeitsmarktpolitischen und sozialen Dienstleistungen zur Integration in Arbeit (Active Inclusion). Übergeordnete Ergebnis- und Leistungsindikatoren, die von der EU-Kommission in Zusammenarbeit mit dem Ausschuss für Sozialschutz entwickelt wurden, sollen im Rahmen eines Benchmarking die Leistungsfähigkeit nationaler Mindestsicherungssysteme auf Basis dieser drei zentralen Prinzipien messen (Bontout und Szatmari, 2020).

## Angemessenheit von Mindestsicherungsleistungen

Leistungen werden auf EU-Ebene als angemessen erachtet, wenn sie in der Lage sind, Armut und soziale Ausgrenzung zu verringern. Gemessen wird dies anhand des Indikators AROPE (At risk of poverty and social exclusion), der sich aus der Armutsgefährdungsschwelle AROP (At risk of poverty), einer geringen Erwerbsintensität auf Haushaltsebene und materieller Entbehrung (Deprivation) zusammensetzt. Die Armutsgefährdungsschwelle AROP, die bei einem (bedarfsgewichteten) Haushaltseinkommen von weniger als 60 % des nationalen Medianeinkommens festgelegt ist, ist in diesem Rahmen das wichtigste Armutsmaß. Dieses relative Armutsmaß hat zwar den großen Vorteil, dass es zwischenstaatlich und intertemporal leicht vergleichbar ist. Es ist aber nicht in der Lage, Verbesserungen der Lebensumstände angemessen abzubilden. So bleibt das Armutsrisiko auch dann konstant, wenn das Wohlstandsniveau aller Mitglieder einer Gesellschaft in gleichem Maße steigt. Zudem sagt AROP nichts über die tatsächliche Unterversorgung mit Gütern (materielle Deprivation) aus. Vielmehr gibt es eine erhebliche Diskrepanz zwischen beiden Risiken. Daten für verschiedene Länder zeigen, dass die beobachteten Trends bei der materiellen Entbehrung oft von den relativen Armutstrends im gleichen Zeitraum abweichen. Dies ist insbesondere in Ländern mit einem relativ hohen Lebensstandard und einer insgesamt geringen materiellen Deprivation wie in Deutschland, den Niederlanden oder den skandinavischen Ländern der Fall.

Der Vergleich der Angemessenheit von Leistungen ist jedoch durch die auf EU-Ebene benutzte Datengrundlage „Leben in Europa“ (EU-SILC) eingeschränkt. EU-SILC ermöglicht keine genaue Identifizierung weder der Empfänger:innen noch der Höhe von Mindesteinkommenstransfers, da diese nicht speziell erfasst werden. In einer jährlichen EU-Haushaltsbefragung werden verschiedene beitragsunabhängige und bedürftigkeitsgeprüfte Leistungen zum Schutz vor „sozialer Ausgrenzung, die nicht anderweitig klassifiziert sind“ als Mindesteinkommen spezifiziert. Diese Transferleistungen sind jedoch nur eine Annäherung an Mindestsicherungsleistungen. Zudem stellen einige Länder keine Informationen zu dieser Variable zur Verfügung bzw. der begrenzte Stichprobenumfang erschwert robuste Ländervergleiche. Schließlich ist die Möglichkeit von Längsschnittanalysen dadurch begrenzt, dass dieselben Personen nur über einen Zeitraum von maximal vier Jahren beobachtet werden können. Dazu kommt, dass für Deutschland die Längsschnittkomponente von EU-SILC bislang überhaupt nicht zur Verfügung stand.

Anders als bei anderen Sozialleistungen ist ein länderübergreifender Vergleich des Leistungsniveaus von Mindestsicherungsleistungen auch deshalb schwierig, weil diese nicht aus einem einzigen Geldbetrag, sondern aus einem ganzen Paket von verschiedenen Geld- und Sachleistungen besteht. Geldleistungen werden in der Regel als Nettobetrag aus Basis- oder Regelleistung, regelmäßigen Zuschlägen (Mehrbedarfe), Wohngeld und Familienleistungen berechnet. Als Soforthilfe werden in vielen Ländern auch zweckgebundene Einzelbedarfe (z. B. für Wohnungsausstattung) gewährt. Regelungen zur Übernahme von anfallenden Wohnkosten existieren in knapp drei Viertel der europäischen Staaten. In einigen Ländern wie z. B. Dänemark oder den Niederlanden sind diese explizit durch die Regelleistungen aber bereits ganz oder anteilig wie z. B. in Österreich (25 % des Regelsatzes) abgedeckt. In Deutschland werden dagegen die tatsächlichen Kosten der Unterkunft (Miete und Heizung) für eine als angemessen definierte Unterkunft übernommen. Sachleistungen können z. B. Gesundheitsleistungen, Nahrungsmittelgutscheine, Schulmittagessen und Sportangebote für Kinder oder verbilligte Fahrkarten etc. umfassen. Das Bildungs- und Teilhabepaket, das Kindern aus Hartz-IV-Haushalten auf Antrag Leistungen zur gesellschaftlichen Teilhabe (Sport, Bildung, Kultur und andere Aktivitäten) zur Verfügung stellt, ist im europäischen Vergleich eine Besonderheit.

Aufgrund der erheblichen Einschränkungen von EU-SILC ist in Abbildung 1 die Höhe der Mindestsicherungsleistungen auf Basis des Tax-Ben-Modells der OECD dargestellt. Das Modell berücksichtigt die komplexen nationalen Re-

Abbildung 1

**Höhe der Mindestsicherung in % des verfügbaren (bedarfsgewichteten) Haushaltseinkommens, 2020**

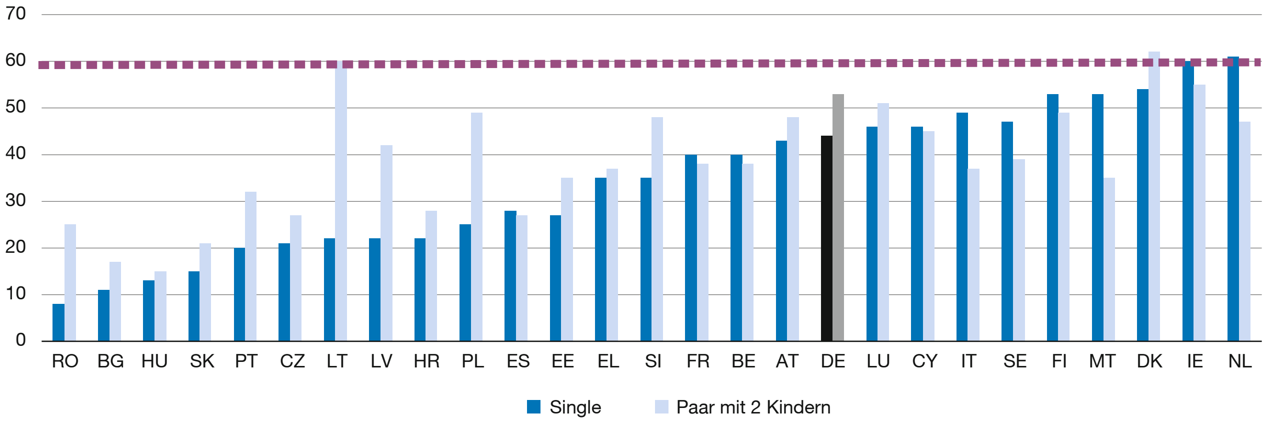

Anmerkungen: Die Berechnungen basieren auf dem Tax-Ben-Modell der OECD. Erfasst sind die wichtigsten Geldleistungen sowie Steuern auf Arbeitseinkommen und Sozialversicherungsbeiträge. Das Wohngeld (Kosten der Unterkunft) wird unter der Annahme berechnet, dass ein Haushalt eine Miete in Höhe von 20 % des Durchschnittslohns zahlt. Die Miethöhe ist für alle Familientypen gleich. Familienleistungen sind in den Berechnungen enthalten. Das verfügbare Medianeinkommen (vor Wohnkosten) wird für das ganze Land berechnet. Die Werte stammen aus nationalen Erhebungen im oder nahe dem Bezugsjahr. Abkürzungen: AT = Österreich, BE = Belgien, BG = Bulgarien, CY = Zypern, CZ = Tschechien, DE = Deutschland, DK = Dänemark, EE = Estland, ES = Spanien, FI = Finnland, FR = Frankreich, EL = Griechenland, HR = Kroatien, HU = Ungarn, IE = Irland, IT = Italien, LT = Litauen, LU = Luxemburg, LV = Lettland, MT = Malta, NL = Niederlande, PL = Polen, PT = Portugal, RO = Rumänien, SE = Schweden, SI= Slowenien, SK = Slowakei.

Quelle: OECD, Adequacy of Guaranteed Minimum Income benefits, https://data.oecd.org/benwage/adequacy-of-minimum-income-benefits.htm.

gelungen, unterstellt aber für eine erleichterte Berechnung über alle Länder hinweg eine Erstattung der Wohnkosten in Höhe von 20 % des jeweiligen Durchschnittslohns.

Abbildung 1 zeigt, dass die deutsche Grundsicherung im Fall von Alleinstehenden im oberen Mittelfeld liegt aber weniger großzügig ist als in Dänemark, Schweden, Finnland, den Niederlanden oder Irland. Bei Paaren mit zwei Kindern liegt die Grundsicherung in Deutschland dagegen im oberen Drittel und bei Alleinerziehenden mit zwei Kindern ist sie mit am großzügigsten in Europa (nicht abgebildet). Dies geht aus Tabelle 1 hervor, in der für ausgewählte Länder die tatsächlich erstatteten Wohnkosten und regelmäßig gewährte Zuschläge kaufkraftbereinigt berechnet wurden. Abbildung 1 zeigt aber auch, dass – mit Ausnahme von Irland und den Niederlanden das Leistungsniveau für Singlehaushalte und in Litauen und Dänemark für Paare mit zwei Kindern – in allen EU-Staaten unter der allgemein definierten Armutsgefährdungsschwelle von 60 % (AROP) liegt und damit als nicht angemessen erachtet wird. Da aber eine beträchtliche Gruppe von Europäer:innen nach dem AROP-Maß von Armut bedroht ist, ohne materiell benachteiligt zu sein, spiegelt AROP eher die Ungleichheit der Einkommen innerhalb einer Gesellschaft als das tatsächliche Armutsrisiko wider.

## Zielgenauigkeit von Mindestsicherungsleistungen

Im EU-Benchmarking gibt es keinen messbaren Indikator für den Zugang zu Mindestsicherungsleistungen. Zwar wurde jüngst der Anteil der Leistungsempfänger:innen unter der armutsgefährdeten Bevölkerung neu in das EU-Benchmarking aufgenommen, es liegen allerdings noch keine vergleichbaren Daten vor. Dieser Indikator spiegelt im Prinzip eher die Zielgenauigkeit in der Armutsbekämpfung wider. Für 2016 zeigen Auswertungen der OECD, dass nur in Deutschland rund 80 % und in Frankreich über 90 % der als einkommensarm definierten Haushalte (mit weniger als 50 % des Medianeinkommens) bedürftigkeitsgeprüfte Leistungen erhalten. Auswertungen aus der IAB-Panelerhebung „Arbeitsmarkt und soziale Sicherung" (PASS) bestätigen die Zahlen für Deutschland. Demnach erhalten 77 % der armutsgefährdeten Personen (mit weniger als 60 % des Medianeinkommens) Arbeitslosengeld II. Unter den nicht auf ALG-II-Leistungen angewiesenen Personen ist der entsprechende Anteil 12 % (Beste et al., 2014). In den osteuropäischen EU-Ländern (Estland, Lettland, Rumänien, Ungarn) und in Südeuropa (Portugal, Spanien) erhielten dagegen weniger als 30 % der einkommensarmen Personen verschiedene bedürftigkeitsgeprüfte Leistungen (Immervoll et al., 2020, Abbildung 1).

Inzwischen hat sich die Situation in Südeuropa aber merklich gebessert. In Griechenland wurde 2017 eine Mindestsicherung nach den oben beschriebenen Prinzipien eingeführt. Eine erste Evaluierung durch die Weltbank 2019 ergab, dass das neue „Sozialeinkommen" 85 % der armen Haushalte erreicht. Auch in Italien (Bürgereinkommen) und in Spanien (Basiseinkommen) wurden in den vergangenen beiden Jahren erstmals landesweite Mindestsicherungseinkommen eingeführt. In den baltischen Staaten und in Polen wurde die Unterstützung insbesondere für Haushalte mit Kindern deutlich ausgeweitet.

Tabelle 1
**Höhe der Mindestsicherungsleistungen in ausgewählten EU-Ländern**
kaufkraftbereinigt in Euro, 2019

| Land | Alleinstehend | Paar mit 2 Kindern (3 und 8 J.) | Alleinerziehend mit 2 Kindern (3 und 8 J.) |
|---|---|---|---|
| Deutschland | 889 | 2.100 | 1.825 |
| Dänemark | 957 | 2.484 | 1.510 |
| Frankreich | 640 | 1.370 | 1.630 |
| Niederlande | 969 | 1.707 | 1.540 |
| Österreich | 861 | 1.550 | 1.386 |

Quelle: eigene Berechnung auf Basis von http://www.oecd.org/els/soc/benefits-and-wages-country-specific-information.htm und https://www.finanz.at/arbeitnehmer/mindestsicherung (Österreich).

## Aktive Eingliederungsleistungen

Wie die deutsche Grundsicherung verfolgen Mindestsicherungssysteme in Europa das Ziel, nicht nur das Existenzminimum der Betroffenen zu gewährleisten. Der von der EU-Kommission favorisierte Active-Inclusion-Ansatz unterstützt einen Aktivierungsansatz im Sinne des Fördern und Forderns. So wird einerseits das Recht auf Zugang zu Arbeitsmarkt und sozialen Dienstleistungen gefordert. Andererseits wird empfohlen, die Gewährung von Mindestsicherungsleistungen an aktive Arbeitssuche, Verfügbarkeit und/oder der Teilnahme an einer Aktivierungsmaßnahme zu knüpfen (Pflichten). Allerdings fehlen im europäischen Benchmarking bislang Indikatoren, die diese Ziele adäquat messen könnten. Das EU-Benchmarking definiert lediglich drei „Hilfs- oder Sekundärindikatoren“ für den Zugang zu Dienstleistungen, die eine aktive Eingliederungspolitik unterstützen und durch Befragungen ermittelt werden: nicht gedeckter Bedarf an medizinischen Untersuchungen, eine Wohnkosten-Überlastungsquote und die Nicht-Teilnahme an berufsbezogener Weiterbildung. Indikatoren, welche die soziale Teilhabemöglichkeiten von Leistungsberechtigten berücksichtigen, fehlen bislang gänzlich. Für ein Benchmarking von Mindestsicherungssystemen wäre es grundsätzlich wichtig zu wissen, ob Aktivitäten wie Kino, Urlaubsreisen, Essen gehen oder Freunde zum Essen einladen bei der Festlegung von monetären Leistungen berücksichtigt werden. Auch hierzulande bestehen in dieser Hinsicht weiterhin Versorgungsdefizite. Eine Studie im Auftrag der EU-Kommission (2018) kommt im Hinblick auf das Angebot von integrierten Dienstleistungen zu dem Ergebnis, dass lediglich in einem Drittel der EU-Länder koordinierte oder vollständig integrierte Sozial- und Beschäftigungsdienstleistungen angeboten werden. Die deutschen Jobcenter zählen zu diesem Drittel.

Zur Messung des Aktivierungsgrads liegen von der OECD entwickelte Indikatoren vor. Sie geben einen Einblick in die Strenge von Aktivierungsanforderungen. Der Gesamtindikator in Abbildung 2 basiert auf der Kombination von Regelungen zu Verfügbarkeit, Arbeitssuche sowie Sanktionen. Demnach liegen die Aktivierungsanforderungen in der deutschen Grundsicherung im europäischen Vergleich im Mittelfeld. Die Sanktionsregelungen im SGB II werden dagegen als vergleichsweise wenig restriktiv bewertet. Im Unterschied zu den De-jure-Regelungen liegen allerdings keine vergleichenden Daten zur tatsächlichen Sanktionspraxis vor.

## Effektivität von Mindestsicherungssystemen

Die Zielsetzung aktivierender Mindestsicherungssysteme ist es nicht nur, Armut durch angemessene Geldleistungen zu vermeiden, sondern auch, erwerbsfähige Personen im Erwerbsalter in existenzsichernde Arbeit zu bringen. Wie erfolgreich die einzelnen EU-Staaten letztlich dabei sind, diese doppelte Zielsetzung zu erreichen, geht aus dem EU-Benchmarking nicht hervor. Die erforderlichen Paneldaten liegen auf EU-Ebene nicht bzw. nur bedingt vor. Eine Alternative wäre nationale Befragungs- und/oder (integrierte) administrative haushalts- und personenbezogene Daten im Längsschnitt zu verwenden. Diese liegen aber nur in einzelnen Ländern vor.

Hierzulande ermöglichen es die IAB-Erhebung PASS (Panel Arbeitsmarkt und soziale Sicherheit) und die Stichprobe der Integrierten Grundsicherungsbiografien (SIG), die Dynamik in der Grundsicherung abzubilden. So zeigen die Auswertungen von Bruckmeier und Co-Autoren (2021) beispielsweise, dass es 2019 1 Mio. weniger erwerbsfähige Beziehende von Grundsicherungsleistungen gab als noch 2010, trotz eines starken Zugangs von Fluchtmigrant:innen in die Grundsicherung nach 2015. Zudem ist nicht nur die Langzeitarbeitslosigkeit unter den ALG-II-Beziehenden stark zurückgegangen, sondern auch die chronische Arbeitslosigkeit, die sich innerhalb von acht Jahren nahezu halbiert hat. Mit dem Konzept der „chronischen Arbeitslosigkeit“ werden auch Personen erfasst, deren Arbeitslosigkeit durch kurze Phasen der Beschäftigung, Inaktivität oder Teilnahme an arbeitsmarktpolitischen Maßnahmen unterbrochen wird und die deshalb nicht zu den offiziellen Langzeitarbeitslosen gezählt werden (zu Konzept und Entwicklung der chronischen Arbeitslosigkeit: IAB-Kurzbericht 1/2018).

Erst die COVID-19-Krise stoppte die positive Entwicklung. Insgesamt ist im Hinblick auf den Rückgang der Zahl der Hartz-IV-Haushalte (Bedarfsgemeinschaften) die Bilanz aber gemischt. Dies ist auch durch den Charakter der deutschen Grundsicherung bedingt. Durch die Integration von Arbeitslosen- und Sozialhilfe in ein umfassendes Sicherungssystem werden Menschen in teils ganz unterschiedlichen Lebenslagen unterstützt. Einerseits ist die Grundsicherung von Gruppen geprägt, die

Abbildung 2

**Strenge der Aktivierungsanforderungen (de jure) in ausgewählten EU-Ländern, 2020**

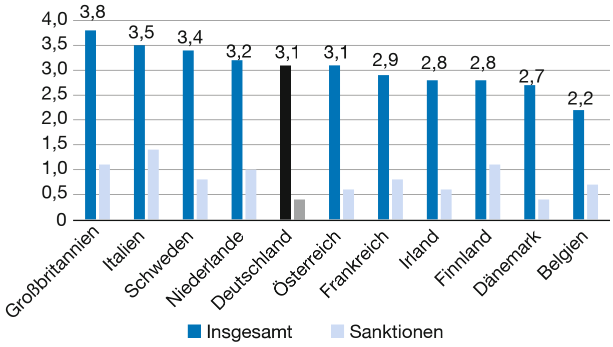

Gesamtscore: 1 (weniger streng) – 5 sehr (sehr streng)

Quelle: http://www.oecd.org/social/strictness-benefit-eligibility.htm.

nur eine temporäre Unterstützung benötigen und andererseits von Bevölkerungsgruppen, die über viele Jahre hinweg im Leistungsbezug verbleiben. So war rund ein Drittel aller Leistungsberechtigten in den vergangenen fünf Jahren durchgehend auf Arbeitslosengeld II angewiesen (Bruckmeier et al., 2021).

## Diskussion um die „Überwindung von Hartz IV“

Angesichts der grundlegenden Bedeutung für die Menschen in Deutschland ist die Ausgestaltung der Grundsicherung für Arbeitsuchende und ihrer Leistungen regelmäßig Gegenstand kontroverser politischer Debatten. Trotz der unbestreitbaren Erfolge des Hartz-IV-Systems wird die Diskussion um den Reformbedarf befeuert durch parteipolitische Forderungen wie, Hartz IV z.B. durch eine Garantiesicherung (Grüne) oder ein Grundeinkommen (Die Linke) zu ersetzen oder zu einem „liberalen Bürgergeld“ (FDP) weiterzuentwickeln. Die Kontroversen reichen von einer Neujustierung des Fördern und Fordern durch Änderungen im Leistungsrecht wie die Neuberechnung der Regelsätze, über ein höheres Schonvermögen, großzügigere Hinzuverdienstregelungen bis hin zur Abschaffung von Sanktionen. So sieht der Vorschlag der Grünen eine bedingungslose aber bedarfsgeprüfte Garantiesicherung vor, welche Existenzsicherung und Aktivierungspolitik entkoppelt. Noch weitergehender sind Forderungen nach einem bedingungslosen Grundeinkommen, mit dem die Grundprämissen der SGB-Grundsicherung, Leistung und Gegenleistung, völlig ad acta gelegt würden.

Auch in anderen Ländern gibt es Diskussionen, bestehende Systeme durch ein leichter zu administrierendes, bedingungsärmeres Grundeinkommen zu ersetzen (Bruckmeier und Konle-Seidl, 2019). Stärker als in anderen Ländern wird die Reformdebatte hierzulande aber unter Gerechtigkeitsaspekten geführt. Der deutsche Sozialstaat Bismarckscher Prägung orientiert sich traditionell stärker am Prinzip der Statussicherung als an der Armutsvermeidung. Daran knüpft sich vielfach die Erwartung, dass Lebensleistung entsprechend honoriert werden sollte. Dies verträgt sich nach Ansicht vieler nicht mit der Tatsache, dass Arbeitslose bereits nach zwölf Monaten in die lohnunabhängige Grundsicherung rutschen und dann jede zumutbare Tätigkeit annehmen müssen. Tatsächlich spielen aber Arbeitslose, die zuvor Arbeitslosengeld I bezogen haben mit einem Anteil von weniger als 10 % an allen Zugängen in die Grundsicherung nur eine geringe Rolle, wie die Statistik der Bundesagentur für Arbeit ausweist. Eine bessere Absicherung langjährig Berufstätiger, z.B. durch eine erhöhte Freistellung von Vermögen bei der Berechnung eines Grundsicherungsanspruchs, wäre als Reformoption aber durchaus denkbar.

Die Grundsicherung hat den durch COVID-19 verursachten Stresstest bestanden. Die Zahl der Bedarfsgemeinschaften ist, anders als zu Beginn der Pandemie vermutet, nicht wesentlich gestiegen. Der im März 2020 mit dem Sozialschutzpaket beschlossene „vereinfachte Zugang zur Grundsicherung für Arbeitsuchende“ kann dabei als eine Art Praxistest für die Weiterentwicklung von Hartz IV genutzt werden. Um niedrigschwellige, unbürokratische Hilfen für bedürftige Erwerbstätigengruppen wie z.B. Solo-Selbständige, Minijobbende und Beschäftigte in Kurzarbeit anzubieten, wurde das Schonvermögen auf 60.000 Euro (Single-Haushalt) ausgedehnt und wurden die tatsächlichen Kosten der Unterkunft (KdU) für einen Zeitraum von sechs Monaten ohne Begrenzung der Wohnungsgröße erstattet. Weiterhin wurde ein Weiterbewilligungsantrag automatisch verlängert, und Sanktionen wurden zwischen März und Mai 2020 gänzlich ausgesetzt und danach gedeckelt. Die Sonderregelungen bei Sanktionen für unter 25-Jährige wurden gemäß dem Urteil des Bundesverfassungsgerichts vom November 2019 abgeschafft.

Eines der erstaunlichsten Ergebnisse einer explorativen Feldstudie im Jobcenter Recklinghausen, die diese temporären Änderungen dort empirisch untersucht, ist die fehlende Akzeptanz für die Abschaffung von Sanktionen nach dem Auslaufen der Corona-Sonderregeln im Dezember 2021. Die Mehrheit (46 % versus 37 %) der befragten Hartz-IV-Beziehenden sprach sich dagegen aus, Sanktionen ganz abzuschaffen. Zudem findet die Feldstudie von Beckmann et al. (2021) eine überraschend hohe Gesamtzufriedenheit von Leistungsbeziehenden sowohl mit dem Jobcenter als auch mit dem Hartz-IV-System insgesamt. Die Autoren kommen weiterhin zu dem Schluss, dass die dauerhafte Vereinfachung bei den Kosten der Unterkunft wie auch die Anhebung der Vermögensgrenze auf 60.000 Euro nur geringe Mehraufwendungen verursachen dürften, zumal die meisten Hartz-IV-Beziehenden über kein nennenswertes Vermögen verfügen – was PASS-Daten bestätigen (Beste et al., 2014).

## Fazit

Eine Stärkung der sozialen Mindestsicherung in Europa ist das erklärte Ziel des kürzlich verabschiedeten Aktionsplans der EU-Kommission zur Umsetzung der Säule sozialer Rechte. In Grundsatz 14 wird das Recht auf „ein angemessenes Mindesteinkommen gefordert, das ein würdevolles Leben und einen wirksamen Zugang zu dafür erforderlichen Gütern und Dienstleistungen“ ermöglichen soll. Damit verbunden ist die Zielsetzung, bis 2030 die Zahl armutsgefährdeter Menschen um 15 Mio. zu verringern. Die EU-Kommission ist aufgefordert, bis Ende 2022 eine Aktualisierung des Unionsrahmens vorzunehmen, um die Politik der Mitgliedstaaten zur nationalen Mindestsicherung wirksam zu unterstützen und zu ergänzen. Auch wenn ein neuer Unionsrahmen wohl eher den Charakter einer Empfehlung als einer verbindlichen Richtlinie haben wird, ist es wichtig, die bestehenden Indikatoren im EU-Benchmarking von Mindestsicherungssystemen zu überarbeiten und zu ergänzen (Konle-Seidl, 2021).

Um auf EU-Ebene die Wirksamkeit von Mindesteinkommenssystemen zu vergleichen, um Orientierung zu geben und die Konvergenz in Europa zu fördern, sind vor allem bessere Daten erforderlich. Die im Zusammenhang mit der Einführung des SGB II aufgebauten Datengrundlagen (wie z.B. PASS oder SIG) können anderen Ländern als „Benchmark“ dienen. Dies gilt auch im Hinblick auf wesentliche Gestaltungsmerkmale der SGB-II-Grundsicherung. Ein breiter Zugang und ein Rechtsanspruch auf Leistungen sind genauso gute Praxis wie das Angebot an integrierten Arbeitsmarkt- und sozialen Dienstleistungen oder das Bildungs- und Teilhabepaket für Kinder in Hartz-IV-Haushalten, das allerdings weniger bürokratisch gestaltet werden sollte.

Hierzulande wirkt der 2005 erfolgte Systembruch „von Bismarck zu Beveridge“ nach und befeuert Reformoptionen bis hin zur einer Abschaffung des Systems („Hartz IV überwinden“). Während Forderungen nach einer Wiedereinführung der alten Arbeitslosenhilfe nur vereinzelt laut werden, haben Forderungen nach einem bedingungslosen Grundeinkommen an Auftrieb gewonnen. Ein bedingungsloses Grundeinkommen ist jedoch angesichts sinkender finanzieller Spielräume weniger denn je zu finanzieren. Würden – ohne Erhöhung der Steuern – alle bestehenden Leistungen für Personen im erwerbsfähigen Alter so ersetzt werden, dass keine Mehrbelastung für die öffentlichen Haushalte entsteht, würde dies nach Berechnungen der OECD (2017) in allen OECD-Ländern zu Leistungsniveaus führen, die unterhalb der Armutsgrenze liegen. Darüber hinaus widerspricht ein bedingungsloses Grundeinkommen nicht nur dem Aktivierungsgedanken, sondern ist auch systemfremd. In einer Erwerbsgesellschaft, in der Erwerbstätige „bedingungslos“ Steuern zahlen (müssen), wird die Gewährung von Leistungen ohne Gegenleistung wohl als unfair erachtet. Ziel sollte deshalb weiterhin sein, mehr Menschen in existenzsichernde Arbeit zu bringen, statt Arbeitslosigkeit über eine Grundsicherung ohne jede Vorbedingung zu finanzieren. Die Überprüfung von Reformoptionen vor dem Hintergrund der Erfahrungen mit der „bedingungsärmeren“ Grundsicherung in Pandemiezeiten wäre dagegen eine überlegenswerte Alternative.

**Title:** ***The German Basic Income System in the European Context***

**JEL Classification:** *I32, I,38, I 39, J08*
